# Preliminary karyotypic data on the Nile pufferfish *Tetraodon lineatus* (Tetraodontidae)

**DOI:** 10.3897/compcytogen.20.212458

**Published:** 2026-10-19

**Authors:** Alexandra Yu. Skuratovskaya, Julia S. Jukova, Fekadu Tefera, Sergey A. Simanovsky, Alexander S. Golubtsov

**Affiliations:** 1 Severtsov Institute of Ecology and Evolution, Russian Academy of Sciences, Leninsky Prospect 33, Moscow, 119071, Russia Severtsov Institute of Ecology and Evolution, Russian Academy of Sciences Moscow Russia; 2 National Fishery and Aquatic Life Research Center, Ethiopian Institute of Agricultural Research, Sebeta, P.O. Box 64, Ethiopia National Fishery and Aquatic Life Research Center, Ethiopian Institute of Agricultural Research Sebeta Ethiopia

**Keywords:** Afrotropical region, chromosomes, cytotaxonomy, Ethiopia, fish cytogenetics, karyology, Tetraodontiformes, White Nile Basin

## Abstract

The family Tetraodontidae comprises 193 valid species of teleost fish distributed in tropical and subtropical regions worldwide. Available cytogenetic information for 31 species indicates extensive variability in diploid chromosome number (2n) ranging from 28 to 46. Here, we present the preliminary cytogenetic characteristics of *Tetraodon lineatus* Linnaeus, 1758, the type species of *Tetraodon* Linnaeus, 1758, which is the type genus of the family. The karyotype of a single male of *T. lineatus* from the White Nile Basin in Ethiopia comprised 12 metacentric/submetacentric and 28 subtelocentric/acrocentric chromosomes (2n = 40, FN = 52). These data are briefly compared with the available cytogenetic information on other tetraodontid genera, summarized according to the current taxonomic classification.

## Introduction

The family Tetraodontidae belongs to the order Tetraodontiformes and comprises 27 genera and 193 species of marine, brackish and freshwater teleost fishes, which are distributed across tropical and subtropical regions worldwide (Fricke et al. 2026). Tetraodontids, commonly known as pufferfishes, are characterized by unique biological traits, including the ability to inflate their bodies, specialized dentition, the production of the potent neurotoxin tetrodotoxin, and compact genomes (Jaillon et al. 2004; Nelson et al. 2016; Froese and Pauly 2026). The genomes of pufferfishes are the most compact among vertebrates (Volff et al. 2003; Jaillon et al. 2004). This reduction is primarily attributed to the contraction of non-coding regions, including introns and intergenic sequences (Brainerd et al. 2001; Neafsey and Palumbi 2003; Jaillon et al. 2004). Nevertheless, the tetraodontid genomes retain structural and regulatory complexity and harbor diverse retrotransposable elements, which may contribute to genome organization and evolutionary dynamics (Volff et al. 2003).

According to the current taxonomic system (Fricke et al. 2026; Table 1), cyto­genetic data are currently available for 31 of the 193 species belonging to nine of the 27 genera. This information shows that tetraodontids are characterized by wide variability in diploid chromosome number from 28 to 46, suggesting notable karyotype differentiation within the family. This is consistent with genomic data revealing variability in structural rearrangements, repeat content and recombination rates among tetraodontids (Kai et al. 2011; Kang et al. 2020; Zhang et al. 2021). In this paper, we report a preliminary karyotype of *Tetraodon lineatus* Linnaeus, 1758, the type species of the genus *Tetraodon* Linnaeus, 1758, based on a single male from the White Nile Basin in Ethiopia. We also briefly summarize the available cytogenetic data on tetraodontids at the genus level according to the most recent taxonomic classification. Tetraodontidae has undergone extensive taxonomic revisions (Kottelat 2013; Araujo et al. 2023; Fricke et al. 2026), and much of the existing cytogenetic data were produced under obsolete systematic arrangements.

**Table 1.** Diploid chromosome numbers (2n) in the genera of the family Tetraodontidae with karyotyped representatives. Some taxa from the original works have been renamed according to their current valid statuses (Fricke et al. 2026). N – number of karyotyped species.

**Genus**	**N**	**2n**	**References**
*Arothron* Müller, 1841	6	38–42	Natarajan and Subrahmanyam 1974; Arai and Nagaiwa 1976; Choudhury et al. 1982; Ojima and Yamamoto 1990; Arai 2011; Reungsing et al. 2020
*Canthigaster* Swainson, 1839	3	28–36	Arai and Nagaiwa 1976; Arai 1983; Martinez et al. 2010; Arai 2011
*Chelonodon* Müller, 1841	2	40	Arai and Nagaiwa 1976; Choudhury et al. 1982; Arai 2011
*Dichotomyctere* Duméril, 1855	2	42	Barat and Khuda-Bukhsh 1984; Fischer et al. 2000; Mandrioli et al. 2000; Mandrioli and Manicardi 2001; Jaillon et al. 2004; Arai 2011
*Lagocephalus* Swainson, 1839	3	44–46	Choudhury et al. 1982; Lakra and Rishi 1991; Sá-Gabriel and Molina 2005; Arai 2011
*Leiodon* Swainson, 1839	1	42	Khuda-Bukhsh and Barat 1987; Arai 2011
*Pao* Kottelat, 2013	2	40–42	Hinegardner and Rosen 1972; Pissaparn et al. 2020
*Sphoeroides* Anonymous, 1798	5	46	Brum et al. 1995; Brum 2000; Sá-Gabriel and Molina 2005; Alves et al. 2008; Vicari et al. 2010; Arai 2011; Noleto et al. 2012; Viana et al. 2017
*Takifugu* Abe, 1949	7	44	Arai and Katsuyama 1973; Arai and Nagaiwa 1976; Arai 1983; Wang and Zhao 1993; Zhao et al. 1994; Miyaki et al. 1995; Yu et al. 1995; Arai 2011; Yoo et al. 2018
*Tetraodon* Linnaeus, 1758	1	40*	This study

* Preliminary data based on a single male.

## Material and methods

Two individuals, both males, of *T. lineatus* (standard length SL = 183 and 214 mm) were obtained from the Alvero River downstream of the Abobo Dam (7°52'23"N, 34°29'48"E) in southwestern Ethiopia. The Alvero River belongs to the Sobat River drainage discharging into the White Nile in South Sudan. *T. lineatus* is the only freshwater tetraodontid known from the Nile Basin (Witte et al. 2009; Fricke et al. 2026; Froese and Pauly 2026). One male was successfully karyotyped.

After colchicine treatment, fish were euthanized with an overdose of tricaine methanesulfonate (MS-222), identified, measured with an accuracy of 1 mm, dissected for gonad examination and tissue sampling, and preserved in 10% formaldehyde. Species identification was carried out based on morphological characters (Golubtsov et al. 1995). The experiments were carried out in accordance with the rules of the Severtsov Institute of Ecology and Evolution (IEE). Vouchers are deposited at the Severtsov Institute of Ecology and Evolution (Moscow), under provisional labels of JERBE.

Before preparation, fish were treated intraperitoneally with 0.1% colchicine (0.01 ml / 1 g of their weight) for 3–5 hours. After euthanasia, chromosome preparations were obtained from kidney and testis tissue following Kligerman and Bloom (1977) with some modifications as described in Simanovsky et al. (2022). The chromosome spreads were stained conventionally with 4% Giemsa solution in a phosphate buffer at pH 6.8 for 8 min. The analysis was performed using an Axioplan 2 Imaging microscope (Carl Zeiss, Germany) equipped with a CV-M4+CL camera (JAI, Japan) and Ikaros software (MetaSystems, Germany). Final images were processed using Photoshop software (Adobe, USA). Karyotypes were arranged according to the centromere position following the nomenclature of Levan et al. (1964), but modified as metacentric/submetacentric (m/sm) and subtelocentric/acrocentric (st/a). Chromosome pairs were arranged according to their size in each chromosome category. To determine the chromosomal arm number per karyotype (fundamental number, FN), metacentrics/submetacentrics were considered as biarmed, and subtelocentrics/acrocentrics as monoarmed chromosomes. The total number of complete mitotic metaphases studied for one *T. lineatus* male was 13 (10 from kidney and 3 from testes).

## Results and discussion

**Figure 1.**
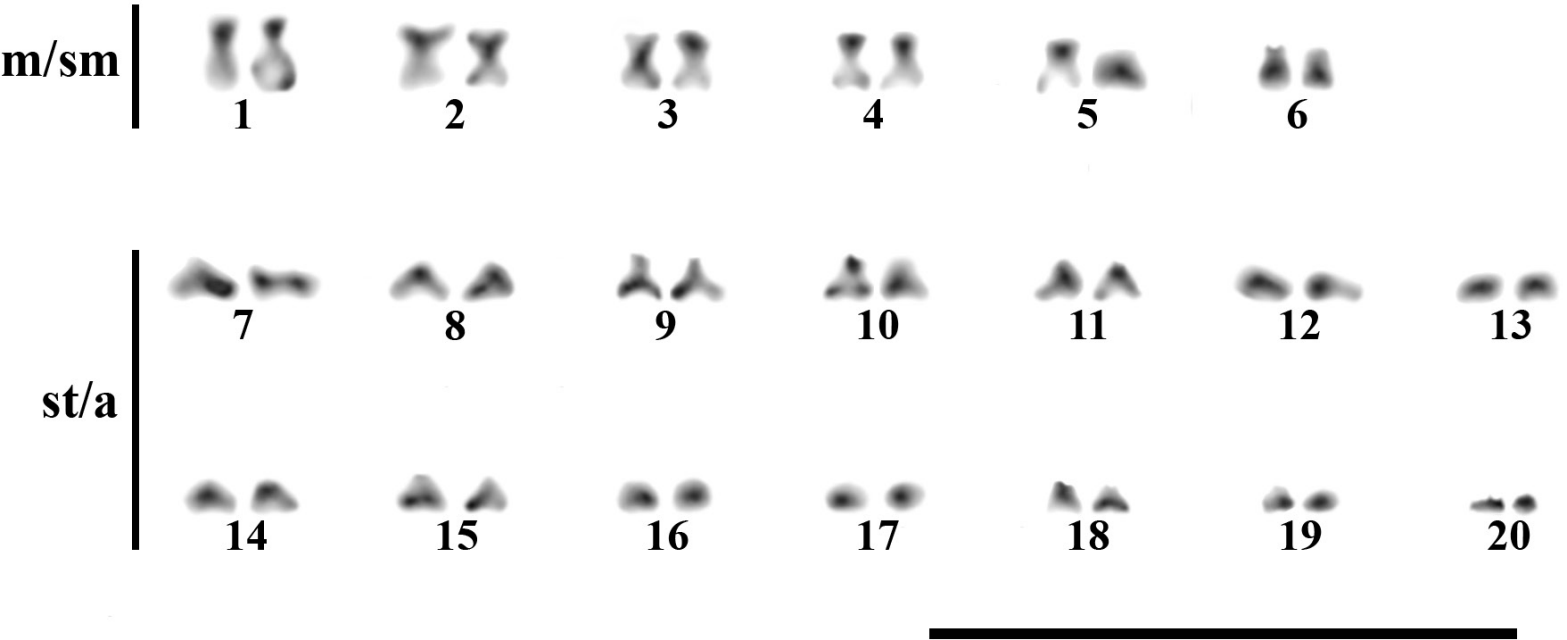
Karyotype of *Tetraodon lineatus* after conventional Giemsa staining. Scale bar: 10 μm.

The examined male *T. lineatus* from the White Nile Basin in Ethiopia has 2n = 40 in all complete metaphases; the karyotype comprises 12 metacentric/submetacentric and 28 subtelocentric/acrocentric chromosomes, FN = 52 (Fig. 1). All chromosomes in this karyotype are very small and difficult to analyze. Other karyotyped members of the family Tetraodontidae exhibit similar characteristics (see references in Table 1). This observation is consistent with the fact that tetraodontids have the smallest genomes among vertebrates (Brainerd et al. 2001; Neafsey and Palumbi 2003; Volff et al. 2003; Jaillon et al. 2004). AgNOR- and C-banding, as well as fluorescent *in situ* hybridization with a (TTAGGG)_4_ telomeric probe, were unsuccessful due to the poor quality of the chromosome material collected under field conditions.

According to the phylogenetic data, Tetraodontidae are divided into two major clades (Santini et al. 2013; Jiang et al. 2018; Cao et al. 2020). We follow the backbone of the multi-gene phylogeny of Santini et al. (2013), noting that the mitogenome-based trees (Jiang et al. 2018; Cao et al. 2020) place several basal taxa differently. Of the genera with karyotyped representatives, Clade 1 comprises only the genus *Takifugu* Abe, 1949. Species of this genus have conserved karyotypes with 2n = 44 (Table 1).

The basal group of Clade 2 comprises the genera *Lagocephalus* Swainson, 1839 and *Sphoeroides* Anonymous, 1798. Members of these genera have karyotypes with 2n = 44–46. The genus *Canthigaster* Swainson, 1839 belongs to the second group within Clade 2. Karyotypes in this genus show extensive variability in 2n, from 28 to 36.

The remaining Tetraodontidae genera with karyotyped members belong to the third group within Clade 2. The karyotyped specimen of *T. lineatus* and the studied species of the genus *Chelonodon* Müller, 1841 share 2n = 40. This uniformity agrees well with the sister-group relationship between *Tetraodon* and *Chelonodon* recovered in all phylogenetic reconstructions (Igarashi et al. 2013; Santini et al. 2013; Jiang et al. 2018; Cao et al. 2020). The genus *Arothron* Müller, 1841, the sister group of *Tetraodon*+*Chelonodon*, has karyotypes with 2n = 38–42. The genera *Pao* Kottelat, 2013, *Leiodon* Swainson, 1839 and *Dichotomyctere* Duméril, 1855 belong to the last group, which is a sister to the group comprising *Arothron* and *Tetraodon*+*Chelonodon*. The karyotyped members of these genera have 2n = 40–42.

Until recently the genus *Tetraodon* included not only African freshwater pufferfishes but also Asian brackish and freshwater representatives (Kottelat 2013; Fricke et al. 2026). Karyotyped members of these Asian groups are now assigned to the genera *Pao*, *Leiodon* and *Dichotomyctere* (Jaillon et al. 2004; Kottelat 2013; Fricke et al. 2026). Our preliminary cytogenetic data on *Tetraodon* are in good agreement with the current taxonomy: *T. lineatus*, as well as representatives of the closely related genus *Chelonodon*, has 2n = 40, whereas all karyotyped members of the genera *Pao*, *Leiodon* and *Dichotomyctere*, with one exception, have 2n = 42.

Given the wide variability in 2n, even conventional karyotyping can provide useful cytotaxonomic markers for tetraodontids. Our study provides a preliminary karyotype of *T. lineatus* from Ethiopia and a baseline for chromosomal studies of African tetraodontids. In general, further cytogenetic studies of Tetraodontidae, integrated with taxo­nomic, phylogenetic and genomic research, are needed. Given that only about 16% of species in the family have been karyotyped to date, our understanding of chromosome diversity within this group remains incomplete.

## Additional information

### Artificial Intelligence (AI) use

The authors accept full responsibility for the content of the manu­script, including the disclosure of any use of AI.

No AI tools were used in the preparation of this manuscript.

### Author ORCIDs

A.Yu. Skuratovskaya https://orcid.org/0009-0000-7404-1161

J.S. Jukova https://orcid.org/0009-0004-8242-8218

S.A. Simanovsky https://orcid.org/0000-0002-0830-7977

A.S. Golubtsov https://orcid.org/0000-0002-8317-7527

### Data availability

All of the data that support the findings of this study are available in the main text.
